# Validity and reliability of the Greek Migraine-Specific Quality of Life Questionnaire (MSQ Version 2.1-GR)

**DOI:** 10.1186/s41687-024-00762-4

**Published:** 2024-07-15

**Authors:** Ermioni Giannouli, Eleni Giannouli, Athanasia Alexoudi, Chryssa Arvaniti, Nikolaos Fakas, Theodoros S. Constantinidis, Evangelos Kouremenos, Dimos-Dimitrios Mitsikostas, on behalf of Hellenic Headache Society

**Affiliations:** 1 Headache Clinic, Athens Medical Centre: Omilos Iatrikou Athinon, 5-7 Distomou Street, Marousi, Athens Greece; 2 1st Psychiatric Clinic, Aeginition Hospital, Medical School, National and Kapodistrian University of Athens, Athens, Greece; 3 First Department of Neurosurgery, Evangelismos Hospital, Medical School, National and Kapodistrian University of Athens, Neurological Institute of Athens, Athens, Greece; 4 Second Department, Medical School, Attikon Hospital, National and Kapodistrian University of Athens, Athens, Greece; 5 Neurology Department, 401 General Military Hospital, Athens, Greece; 6 Neurology Clinic, Korinthos, Greece; 7 Neurology Department, 251 General Hospital of Air Forces, Athens, Greece; 8 First Department of Neurology, Medical School, Aeginition Hospital, National and Kapodistrian University of Athens, Athens, Greece

**Keywords:** Migraine-specific quality of life, Migraine, Psychometric properties, Validity, Migraine-Specific Quality of Life Questionnaire

## Abstract

**Background:**

To assess the validity and reliability of the Migraine-Specific Quality of Life Questionnaire 2.1 (MSQv.2.1) in a group of Greek migraineurs.

**Design—sample—methods:**

The Greek version of MSQv.2.1 (MSQv.2.1-GR), a self-report measure with 14 items in 3 domains (Role Restrictive (RR), Role Preventive (RP) and Emotional Function (EF)), was administered during a cross-sectional study to 141 Greek adult migraineurs and 135 controls without migraine or any other primary headache disorder, along with Migraine Disability Assessment Scale (MIDAS) and Short Form Health Survey (SF-12) to assess validity. MSQv.2.1-GR was re-administered in a group of participants with migraine two weeks afterwards to assess reliability. Content and construct validity was assessed using Intraclass Correlation Coefficient (ICC), Spearman rho, McDonald’s omega, Cronbach’s alpha. Confirmatory factor analysis (CFA) was used to test the latent structure of the MSQv.2.1-GR in migraineurs.

**Results:**

A total of 276 adults participated in the study. Internal consistency of the three MSQv.2.1-GR scales RR, RP and EF yielded a range of McDonald’s omega from 0.832 to 0.923 (Cronbach’s alpha from 0.814 to 0.923). CFA confirmed the proposed three-factor MSQv.2.1-GR latent structure with acceptable goodness of fit indices and factor loadings. Correlations were established between MSQv2.1-GR component and MIDAS scores, showing moderate and statistically significant relationships (from − 0.519 to −0.562, all *p* < 0.001) for RR, RP and EF. Correlations between MSQv2.1-GR and SF-12 component scores were identified, with values from 0.1 to 0.4, indicating low to moderate associations. ICC was calculated at 0.997, indicating a high level of reliability between the measures. Notably, all MSQv2.1-GR scores (RR, RP, EF) were significantly higher in the controls compared to migraineurs (*p* < 0.001 for all scales). These findings suggest that MSQv2.1-GR is internally consistent, shows significant correlations with relevant measures, and is effective in discriminating controls from migraineurs.

**Conclusion:**

MSQv2.1-GR is a valid and reliable tool to determine the effect migraine has on the quality of life of Greek-speaking migraineurs.

**Supplementary Information:**

The online version contains supplementary material available at 10.1186/s41687-024-00762-4.

## Abstract (plain language summary)

Migraine is a major global health issue, ranking at the top leading causes of disability worldwide. The Migraine-Specific Quality of Life Questionnaire 2.1 (MSQ v.2.10 is an effective instrument for measuring the influence of headaches on a patient’s quality of life but it is not available for the Greek migraineurs. We therefore made this study to investigate the reliability and validity of MSQv.2.1 in Greek. The Greek version (MSQv2.1-GR) was administered on a total of 142 migraineurs and 136 non-migraineurs. In addition, other instruments such as MIDAS and SF-12 were applied to determine its validity. We concluded that MSQv2.1-GR is a valid measure. There was great reliability between the items because they were measuring the same thing. It had a good correlation with other tests that evaluate migraine-related disability and quality of life. It also differentiated migraineurs from non-migraineurs in terms of quality of life. Therefore, MSQv2.1-GR proves to be an appropriate measure for examining the consequences of migraine on the health-related quality of life among Greek individuals. This tool will be helpful for clinicians in Greece as well as for research purposes such as cross-cultural studies on effects of migraine on quality of life.

**Supplementary Information:**

The online version contains supplementary material available at 10.1186/s41687-024-00762-4.

## Introduction

Migraine is a primary headache disorder affecting more than 1 billion people worldwide [1, 2]. It is one of the five leading causes of disability worldwide, with women being affected much more than men [3]. In the 2019 Global Burden of Disease iteration, migraine alone was second among the causes of disability, and first among women under 50 years of age [4–6]. The disability related to migraine affects the patients’ personal life (interpersonal relationships, family relationships with partners and children), as well as their work status (days of absence from work, which account for a financial toll in the region of billions in the United States) [7–9]. The commonality of the disease, the significant impact on patients’ personal lives and the immense financial burden make migraine a significant contributor to the global burden of disease. Measuring this burden is, therefore, of paramount importance and shows how migraine affects the individual patient as well as the possible improvement certain treatments can provide over time.

Using general health status instruments like SF-36, a number of studies determined that migraineurs have greater impairment in certain health-related quality of life (HRQOL) dimensions compared with patients suffering from other chronic diseases. In addition, general measures facilitate comparisons across patient populations and disease states, but they are less sensitive to changes in HRQOL within the context of a clinical intervention. Hence, it was deemed important to develop disease-specific instruments like MSQv2.1 that measure functional limitations and restrictions associated with a specific disease state [10].

The Migraine-Specific Quality-of-Life Questionnaire Version 2.1 (MSQ v.2.1) is a self-report measure that assesses the impact of migraine on three domains of quality of life: role functioning, emotional functioning, and social functioning. The MSQ v.2.1 consists of 14 items that are rated on a 6-point Likert scale, ranging from 1 (very poor) to 6 (very good). The MSQ v.2.1 has been widely used and proven effective in various languages and population samples [11–17].

In the original validation study of the MSQ v.2.1 by Martin et al. [11], a sample of 373 American migraineurs were asked to provide information concerning their migraines, its severity, effect on health, and social life by completing the MSQ v.2.1. The study revealed adequate psychometric properties of the MSQv.2.1 including, high internal consistency (Cronbach’s alpha = 0.93), test-retest reliability (intraclass correlation coefficient = 0.80), convergent validation (correlation with Migraine Disability Assessment Scale = −0.71), discriminant validity (correlation with Short Form-36 Health Survey = 0.54), and criterion validity (correlation with headache frequency = −0.40). The factor structure of the MSQ v.2.1 was also confirmed by exploratory (EFA) and confirmatory factor analyses (CFA), with three factors corresponding to the three domains of quality of life: role restrictive, role preventive and emotional function.

Several studies have since replicated and extended the validation of the MSQ v.2.1 [12, 18] in different languages and population samples, such as Chinese [12, 13] and Persian [14], among others. Overall, these studies have shown that the MSQ v.2.1 is reliable, valid, and factors can be used to develop gender-specific, age, education, and regional specific norms for different cultural contexts and settings. On the other hand, it should be noted that there have been other reports with respect to the MSQ v.2.1 showing some limitations or challenges, such as cultural differences in response styles, ceiling effects in some items or domains, sensitivity to change over time or treatment, and applicability to different types or subtypes of migraine [19].

As mentioned above, MSQ v.2.1 has shown excellent validity to both episodic and chronic migraineurs [15–17] but, to date, there is no validated version of the MSQ v.2.1 in the Greek language, which limits its use for research and clinical purposes in Greece. We aimed to assess the validity and reliability of MSQ v.2.1 in a sample of Greek-speaking migraineurs with episodic and chronic migraine, with and without aura.

## General methods

### Study design, setting and participants

This cross-sectional study employed a consecutive sampling approach within the authors’ neurological outpatient practices in Athens and Korinthos, Greece, between March and September 2023.

The inclusion criterion was a diagnosis of migraine with or without aura, episodic or chronic, made by all participating neurologists who are experts in the field, based on the 2018 International Headache Society criteria (3rd edition) [20]. Exclusion criteria encompassed individuals who were younger than 18 years of age (no upper age limit was defined in the study), infrequently experienced migraines, or had a diagnosis of other primary headache disorder other than migraine. Eligible patients were adults with a good understanding of the Greek language who came to the clinic during the recruitment period. Present or past antimigraine treatments were not recorded. The research team explained the study’s objectives, and interested patients were requested to provide written informed consent.

The controls were patients over 18 years old, visiting each participating neurologist’s office or clinic, who did not suffer from migraine or any other primary headache disorders. The controls were informed about the study, any questions were answered, and the interested patients provided written informed consent before participating.

Mapi Research Trust^®^ provided us with the Greek translation of the MSQv.2.1 questionnaire. One of the authors administered a pilot administration (*N* = 15) where one researcher administered the questionnaire. Participants in this study were asked a series of questions, including whether they found the questionnaire easy to understand, if they had any difficulties in completing it, and any other problems they may have encountered. No difficulties were reported, and no problems were identified, indicating that the MSQv2.1-GR is easy to understand and complete.

According to COSMIN recommendations which proposes at least a sample size of 100 [21] and since other investigators suggest a range number from 2 to 20 responders for each item of the questionnaire [22] with an absolute minimum of 100 to 250 subjects [23, 24] authors decided a minimum number of 250 participants.

The patients and matched controls completed a predefined demographic data form (which included gender, age, educational level, work status, marital status along with basic data about migraine, namely attack frequency, presence or absence of aura), the Greek version of MSQ v2.1, as well as the Greek versions of MIDAS and SF-12 questionnaires, already validated in the Greek language [25], to determine validity. Each participating neurologist administered the questionnaires in their respecting offices. The participants were provided with the necessary instructions on how to fulfill the questionnaires and all answers were collected using paper-based questionnaires.

A group of participants (*n* = 35) were then re-administered MSQv2.1-GR two weeks after the initial administration to assess test-retest reliability (stability). The same interviewer administered the questionnaires each time. The optimal time-interval between testing varies depending on the construct being measured, on the stability of the construct over time and the target population, however, the target time of 2 weeks is the most frequently recommended interval [26].

Data was collected to assess CFA, internal consistency, convergent validity, differences between groups and test-retest reliability of the Greek MSQ v2.1.

### Instruments

#### Migraine-Specific Quality of Life Questionnaire version 2.1 (MSQv2.1)

MSQv2.1 is a self-report measure with a total of 14 items which assesses the impact of migraine on three quality of life domains, during the last 4 weeks: (a) *Role Restrictive (RR)*, which includes 7 items that measure the functional impact of migraine, meaning how migraine limits the patient’s performance in everyday work and social activities, (b) *Role Preventive (RP)*, which consists of 4 items that measure how migraine interrupts a patient’s everyday social and work activities, and (c) *Emotion Function (EF)*, with 3 items which assess the impact of migraine on the responder’s emotions (e.g. frustration or despair). The item responses range from one to six (1=“None of the time;”2=“A little bit of time;”3= “Some of the time;” 4 = “A good bit of the time;” 5 = “Most of the time;” 6 = “All of the time”). All items are reverse-coded, and standardized to a 0–100 scale. Thus, higher scale scores indicate better migraine-related quality of life [11]. In the development study [11] the internal consistency coefficients ranged from 0.86 to 0.96, and the intraclass correlation coefficients ranged from 0.57 to 0.63 across the three dimensions. As anticipated, the MSQ dimensions had low-to-modest correlations with the two component scores of the SF-36 and were modestly to moderately correlated with migraine symptoms.

#### Migraine Disability Assessment Scale (MIDAS)

The assessment of migraine-related disability utilized the Migraine Disability Assessment Scale (MIDAS), which consists of 5 items. Over a reference period of the past 3 months, the MIDAS evaluates individuals’ disability in three domains: work/school, household responsibilities, and social activities. The total MIDAS score has a potential range from 0 to 270 and is categorized into levels of disability as follows: (1) 0 to 5 indicating slight or minimal disability, (2) 6 to 10 signifying low levels of disability, (3) 11 to 20 representing moderate disability, and (4) 21 or higher indicating severe disability. The MIDAS has demonstrated satisfactory test-retest reliability over a 21-day period, with Spearman’s rho values ranging from 0.67 to 0.73 for individual items and strong test-retest reliability (Spearman’s rho = 0.84) for overall scores. Furthermore, it has shown good internal consistency, with a Cronbach’s α value of 0.83. In addition, MIDAS scores have displayed a moderate correlation with pain intensity scores derived from a pain diary (*r* = 0.63), thus confirming its convergent validity. Lastly, the MIDAS scores have proven to be significantly distinguishable between individuals with migraines and those without, supporting its discriminant validity [11, 27–29]. Our hypothesis was that lower MSQv2.1-GR scores i.e. greater burden in quality of life would be correlated with higher MIDAS scores, i.e. more severe disability.

#### Short Form Health Survey (SF-12)

To evaluate the convergent validity of the Greek version of the MSQv2.1, all participants underwent an assessment using the 12-Item Short Form Health Survey (SF-12), which measures various functional aspects. The original SF-36 represents all the dimensions in question. The SF-12 [26] is a concise adaptation of the SF-36 and has been demonstrated to effectively represent the original SF-36 scores [30]. The SF-12 assesses eight function domains: physical functioning, role-physical, role-emotional, mental health, bodily pain, general health, vitality, and social functioning. These eight domains can be summarized into two primary summary scores assessing physical functioning (i.e. physical component summary score, PCS) and psychological functioning (i.e., a mental component summary, MCS). The SF-12 exhibits robust psychometric properties, including strong test-retest reliability and validity across a range of patient populations [26, 30–32]. To evaluate the convergent validity of the Greek version of the MSQv2.1, all participants underwent an assessment using the SF-12. The hypothesis was that if MSQv2.1-GR was valid, it would be significantly associated with the SF-12 subscales.

### Statistical analysis

All statistical analyses were conducted using statistical software IBM© SPSS© version 25 (IBM Statistical Package for Social Sciences for Windows, Version 25.0. Armonk, NY: IBM Corp) and R Statistics software version 4.0.3 [33] using the Lavaan package (Yves Rosseel (2012). lavaan: An R Package for Structural Equation Modeling.). The COSMIN guidelines were used for reporting and analyzing the results [34]. The statistically significant level was set at 0.05.

Confirmatory factor analysis (CFA) using maximum likelihood estimator was evaluated using the comparative fit index (CFI), Tucker-Lewis index (TLI) and the root mean square error of approximation (RMSEA) with 95% Confidence Interval (CI). Internal consistency was evaluated using McDonald’s omega coefficient and Cronbach’s alpha, convergent validity using Spearman rho correlation coefficient, stability (test-retest) using Intraclass Correlation Coefficient (ICC) with a two-way random effects model and absolute agreement. Correlations from 0.1 to 0.39 are characterized as low and 0.4 to 0.69 as moderate [35]. Authors used the next cutoff points to evaluate good model fit: (i) an RMSEA in the range of 0.05 to 0.10 was considered an indication of acceptable fit and values above 0.10 indicated poor fit [36] (ii) a value of CFI ≥ 0.95 is accepted as indicative of good fit [37], (iii) TLI ≥ 0.95 is a cutoff criterion that frequently used for the goodness of fit [38, 39]. Model fit was also evaluated using the minimum fit function χ^2^. As χ^2^ values are usually inflated by large sample sizes, we also evaluated the χ^2^/df ratio, where a value of 5 or less is assumed as acceptable (MacCallum, Brown, & Sugawara, 1996). Non- parametric Mann-Whitney U test was used in order to check any differences between two groups (migraineurs vs. controls) while chi-square test was used in quantitative variables. For dealing missing data, while (i) adequate large sample was ensured and (ii) the assumption of missing data completely at random is satisfied, the listwise deletion was decided as the optimum dealing with as the most frequently used method in handling missing data in most statistical software packages. While the aforementioned assumptions were covered, the bias in the estimation of the parameters was negligible.

### Ethical considerations

The study was approved by the Institutional Review Board of Eginition University Hospital bearing the approval number ΑΔΑ: 63ΧΠ46Ψ8Ν2–43Φ and conducted under the auspices of the Hellenic Headache Society. Prior to collecting data, we obtained consent in writing from each participant. The individuals who agreed willingly, were the subjects asked to fill in the study questionnaires. Later on, a portion of them were requested at the end of two weeks for repeat interviews on test-retest reliability using the MSQv2.1-GR once more. All data were anonymized.

## General results

### Demographic and other characteristics

A total of 278 subjects (142 migraineurs and 136 controls) who met inclusion and exclusion criteria were recruited in the present study. Two participants were excluded due to missing data using listwise deletion decreasing the final sample to 276 subjects (141 migraineurs and 135 controls). The majority of the patient sample consisted of females (68.8%; total sample) while there were more males in the control group compared to the migraineur group (43.4% vs. 19.3% in cases, *p* < 0.001). Age was also, dependent on the group (migraineurs vs. controls), statistically significant (*p* = 0.005). Family status, children and educational level were not significantly different in the two groups (*p* = 0.148, *p* = 0.052, *p* = 0.539). While medical issues were not significantly different in the two groups (*p* = 0.113), mental health problems were found to be different between the two groups (*p* = 0.006) i.e., migraineurs had mental health issues at a higher frequency compared to controls (19.4% and 8.1%, respectively) (Table 1).

**Table 1:** Demographic and other characteristics separated by patients and controls

	All (*N* = 276)	Patients (*n* = 141)	Controls (*n* = 135)	*p*
**Gender**				
Male	84 (30.7)	26 (18.7)	58 (43.0)	**< 0.001**
Female	190 (69.3)	113 (81.3)	77 (57.0)	
**Age (years)**				
18–24	16 (6.3)	5 (3.9)	11 (8.9)	**0.004**
25–32	62 (24.5)	26 (20.2)	36 (29.0)	
33–40	4 (17.4)	29 (22.5)	15 (12.1)	
41–48	53 (20.9)	34 (26.4)	19 (15.3)	
49–56	34 (13.4)	16 (12.4)	18 (14.5)	
56–61	20 (7.9)	7 (5.4)	13 (10.5)	
62–66	14 (5.5)	10 (7.8)	4 (3.2)	
67+	10 (4.0)	2 (1.6)	8 (6.5)	
**Family status**				
Unmarried	116 (43.3)	52 (38.2)	64 (48.5)	0.179
Married	131 (48.9)	74 (54.4)	57 (43.2)	
Divorced	21 (7.8)	10 (7.4)	11 (8.3)	
**Children**				
Yes	105 (57.1)	69 (62.7)	36 (48.6)	0.059
No	79 (42.9)	41 (37.3)	38 (51.4)	
**Educational level**				
Till secondary education	78 (29.1)	37 (27.4)	41 (30.8)	0.538
Higher education	190 (70.9)	98 (72.6)	92 (69.2)	
**Medical issues**				
Yes	129 (47.4)	72 (52.2)	57 (42.5)	0.112
No	143 (52.6)	66 (47.8)	77 (57.5)	
**Mental health problems**				
Yes	37 (13.6)	26 (18.8)	11 (8.1)	**0.010**
No	236 (86.4)	112 (81.2)	124 (91.9)	

*Notes* Values are referred to absolute and relative frequencies (%). *P*-value is computed using chi-square test

### Pre-testing and pilot study

Pre-testing was conducted to check the face validity which was established by two expert neurologists in this field. They did not report any non relevant items in the questionnaire. Also, a pilot study was conducted using 15 migraineurs. The overall McDonald’s omega was found as 0.952 (Cronbach’s alpha 0.985). Moreover, patients from this pilot study were asked for difficulties that they faced or any other problems that they may have encountered. No difficulties were reported.

### Confirmatory factor analysis

Confirmatory factor analysis was assessed in the subsample with the migraineurs (Table 2). Factor loadings indicated an adequate support for the three-factor model of the MSQ as recommended from the authors of the original tool while all factor loadings were above 0.70, except for item 12 (‘‘have you felt fed up or frustrated because of your migraines?’’). The factor loading for this item (frustrated) was computed as 0.57.

The goodness of fit indices also suggested that the investigated three-factor model was an adequate representation of the latent structure. The RMSEA (90% CI) was found as 0.10 (0.08–0.12); CFI as 0.95; TLI as 0.94. In this CFA, χ^2^/df ratio was found as 2.36.

Mean of all items was ranged from 2.21 to 3.75.

The overall internal consistency was computed after conducting McDonalds’s omega coefficient which was found as 0.951 (Cronbach’s alpha 0.952). McDonalds’s omega for each scale was found as 0.918, 0.923 and 0.832 for Role Restrictive (RR), Role Preventive (RP) and Emotional Function (EF), respectively (Cronbach’s alpha 0.917, 0.923 and 0.814 respectively). No significant floor or ceiling effects were observed for any of the three domains.

**Table 2:** Confirmatory factor analysis for MSQv2.1-GR: item descriptive statistics, standardized factor loadings, parameter estimates and internal consistency

Item	Label	Mean ± SD	Standardized estimates of factor loadings	Estimates of factor loadings (SE)	McDonald’s omega
Role Restrictive (RR)					0.918
MSQ1	Family	3.11 ± 2.10	0.80	1.07 (0.10)	
MSQ2	Leisure	3.31 ± 1.31	0.88	1.15 (0.09)	
MSQ3	Activity	3.16 ± 1.34	0.93	1.25 (0.09)	
MSQ4	Work	3.13 ± 1.25	0.92	1.15 (0.08)	
MSQ5	Contract	3.19 ± 1.33	0.92	1.22 (0.09)	
MSQ6	Tired	2.83 ± 1.38	0.85	1.17 (0.10)	
MSQ7	Energy	3.16 ± 1.41	0.84	1.18 (0.10)	
Role Preventive (RP)					0.923
MSQ8	Cancel	2.31 ± 1.29	0.90	1.16 (0.09)	
MSQ9	Help	2.42 ± 1.33	0.85	1.12 (0.09)	
MSQ10	Stop	2.62 ± 1.33	0.86	1.16 (0.09)	
MSQ11	Social	2.55 ± 1.39	0.89	1.24 (0.100	
Emotional Function (EF)					0.832
MSQ12	Frustrat	3.75 ± 1.73	0.57	0.98 (0.14)	
MSQ13	Burden	2.21 ± 1.57	0.96	1.51 (0.11)	
MSQ14	Afraid	2.28 ± 1.60	0.88	1.41 (0.11)	
Chi-square (DF)	174.29 (74)				
CFI/TLI	0.95/0.94				
RMSEA (90% CI)	0.10 (0.08–0.12)				

*Abbreviations SD* standard deviation, *SE* Standard Error

### Convergent validity

Evidence of convergent validity was supported by the Spearman rho correlation coefficient between MSQv2.1-GR dimension scores, SF-12 scores and MIDAS scores (Table 3). As hypothesized, the correlations between MSQv2.1-GR component scores and MIDAS total score were moderate and statistically significant (*r* = −0.562, *r* = −0.542, *r* = −0.519, respectively for RR, PR, EF, *p* < 0.001, effect size d = 0.3 for all which indicates small effect [39].

The correlations between MSQv2.1-GR component scores and SF-12 component scores were low to moderate (ranged from 0.1 to 0.4). Scores correlated between MSQv2.1-GR dimension scores with MCS were larger than the correlations with PCS component.

**Table 3:** Convergent validity: MSQv2.1-GR, MIDAS and SF-12 Spearman rho correlation coefficients

Scales	RR	PR	EF	MIDAS	SF-12 PCS
Role Restrictive (RR)	1				
Role Preventive (RP)	0.840***	1			
Emotional Function (EF)	0.714***	0.752***	1		
MIDAS	−0.562***	−0.542***	−0.519***	1	
SF-12 PCS	0.295***	0.195*	0.134	−0.271**	1
SF-12 MCS	0.376***	0.291**	0.280**	−0.261**	−0.068

*Abbreviations SF-12* the 12-item Health Survey, *PCS* Physical Component Summary, *MCS* Mental Component Summary, *MIDAS* Migraine Disability Assessment Questionnaire

***p < 0.001, **p < 0.01, *p < 0.05

### Test-retest reliability coefficient

Test-retest reliability was conducted in order to check the measure of reliability obtained by administering the same tool (MSQv2.1-GR) twice after 2 weeks from the initial administration to a group of migraineurs (*n* = 34). ICC was found as 0.997 (95% confidence interval 0.993–0.998, *p* < 0.001). ICC values < 0.5 indicate poor reliability, values from 0.5 to 0.75 indicate moderate reliability, 0.75–0.9 good reliability, and values > 0.90 indicate excellent reliability (Portney LG, Watkins MP. Prentice Hall; New Jersey: 2000. Foundations of clinical research: applications to practice.) Our results fall in the category of excellent reliability indicating stability among the participants.

### Differences between groups

All MSQv2.1-GR dimension scores (Table 4) were significantly higher for controls compared to migraineurs (*p* < 0.001 for all scales). This pattern was evidenced in MIDAS scale as well (*p* < 0.001) where patients were assessed with higher migraine disability (Mdn = 26) compared to controls (Mdn = 0). PCS from SF-12 was found with a non-significant difference between groups while MCS from the same tool (*p* < 0.001) was statistically higher in controls (Mdn = 50.46) compared to migraineurs (Mdn = 44.11).

**Table 4:** MSQv2.1-GR, MIDAS and SF-12 component scores compared to patients’ and controls’ subgroups

Scales/components	Patients	Controls	U	*p*
Role Restrictive (RR)	60.00 (37.14)	100.00 (0.00)	1222.00	**< 0.001**
Role Preventive (RP)	75.00 (35.00)	100.00 (0.00)	2106.50	**< 0.001**
Emotional Function (EF)	66.67 (40.00)	100.00 (0.00)	1773.00	**< 0.001**
MIDAS	26.00 (37.00)	0.00 (0.00)	637.50	**< 0.001**
SF-12 PCS	43.50 (9.59)	44.28 (5.37)	8380.00	0.774
SF-12 MCS	44.11 (16.72)	50.46 (11.99)	5332.00	**< 0.001**

*Abbreviations SF-12* the 12-item Health Survey, *PCS* Physical Component Summary, *MCS* Mental Component Summary, *MIDAS* Migraine Disability Assessment Questionnaire

*Notes* The values are referred to median and interquartile range using Mann-Whitney U test. Statistically significant differences are indicated in bold

## Discussion

The MSQv2.1 is a valid assessment tool for migraine-related quality of life which has proven invaluable in determining the impact migraine has on the individual patient and it can also be used to monitor the effect certain treatments or interventions may have on the patient both when they are initially administered and as a monitoring tool for their continued response. Consequently, MSQv2.1 has served as a fundamental research objective in many clinical trials, where the improvement in its score clearly correlates with a decreased disease burden and is instrumental in determining the effectiveness of the drug being studied [40–42].

To our knowledge, this is the first study conducted in a Greek population which aims to assess the psychometric properties of the Greek version of MSQv2.1, assuming they would be similar to other validity studies of this questionnaire. In doing so, we sought to provide an important tool for clinicians and researchers in Greece, enhancing their ability to evaluate migraine-related quality of life.

Demographics were similar to other validation studies which report a higher prevalence of migraine in females, between the ages of 25–49 years old.

Participants in our study do not represent a specific migraine population but rather an assortment of different migraine patients (episodic and chronic migraine, with and without aura, low and high disease burden, low and highly educated people), being more representative of the wide array of patients seen in headache clinics. While there are many studies [15–19] which prove MSQv.2.1 reliability in either episodic or chronic migraine, in the present study we did not assess the different migraine populations separately.

Expert neurologists as well as participants in the pilot study did not report any difficulties in understanding or administering the Greek version of the MSQ v2.1 proving that the Greek version of the questionnaire is a well-understood and easy to administer tool.

The MSQv2.1-GR demonstrated high internal consistency (McDonalds’s omega coefficient at 0.951 whereas McDonalds’s omega for each scale was found as 0.918, 0.923 and 0.832 for Role Restrictive (RR), Role Preventive (RP) and Emotional Function (EF), respectively). Test-retest reliability was also commendable since ICC was found to be 0.997 (95% confidence interval 0.993–0.998, *p* < 0.001). These findings align with prior reports that have underscored the reliability of the English version of the questionnaire [11, 19]. Our observation that the correlations between MSQv2.1 and SF-12 (ranging from 0.1 to 0.4) are in line with the range of correlations documented in the literature (which typically fall between 0.19 and 0.38 [11–17, 19]), provides support for the convergent validity of the MSQv2.1-GR. Furthermore, the negative correlations (high MIDAS score with low MSQv2.1 score) identified between MIDAS, and MSQv2.1 were moderate and statistically significant (*r* = −0.562, *r* = −0.542, *r* = −0.519, respectively for RR, PR, EF, *p* < 0.001) and correspond with findings in the literature (ranging from − 0.57 to −0.10) [10–16], thereby reinforcing the criterion validity of the Greek version.

In our study, no significant correlation was found between PCS component of SF-12 and MSQv2.1 scores in the migraineurs which is in contradiction to other studies [14], but a significant correlation was found between MCS component of the SF-12 and MSQv2.1. Since our demographic data show that in our sample mental health issues were more prominent in the migraineurs compared to controls, it stands to reason that these people would report more problems with low energy, lack of peace and calmness, or feelings of frustration and despair (measured in the MCS component of SF-12). On the other hand, seeing that in our study the control group was found to score higher in the MCS component of the SF-12 compared to migraineurs which could be an indicator of psychiatric disorders or other comorbidities, the fact that these participants also have a high score in MSQv2.1 (better quality of life) further strengthens the validity and reliability of MSQv2.1 as a measure of quality of life specific to migraine and not a general measure of quality of life.

The results affirm the reliability and validity of MSQv2.1-GR in appraising the quality of life specific to individuals with migraine. These findings make a valuable contribution to the MSQv2.1 literature by providing a valuable tool for the Greek population, thereby expanding our capacity to evaluate the quality of life among a more extensive group of migraineurs. Additionally, the availability of a Greek rendition of the MSQ opens up the potential for cross-cultural research investigating the quality of life of individuals dealing with migraine headaches.

## Limitations and future research

Several significant limitations should be taken into account when interpreting the study’s outcomes. The high internal consistency coefficient in both the pilot study and overall tool could be included. Generally, a high value of internal consistency coefficient (> 0.90) may suggest redundancies and the test length should be shortened. We have checked the length of the questionnaire, and it was found optimal, along with the internal consistency in other studies which was found satisfactory. Also, we checked for redundancy and found that there were no items with different semantics which measured a similar characteristic of the latent trait. The correlation between items also was analyzed and no high correlation was revealed. Three assessments were considered; (i) the internal consistency coefficient was reduced from the pilot study to the main research, (ii) the indices of the subscales were at tolerable levels, and (iii) two other validations were found with high internal consistency coefficient, which led us to accept the internal consistency coefficient as is.

Another limitation of the study is that it did not assess the MSQv2.1-GR’s sensitivity to changes following treatment, a crucial aspect of validity, particularly when the measure is intended for assessing clinical trial outcomes. Future research should address this essential aspect of validity.

In addition, all measures employed in this study were self-reported, which can potentially intensify the relationships observed due to method variance. Therefore, future research focusing on the psychometric properties of the MSQv2.1-GR should endeavor to incorporate objective measures of criterion variables when feasible.

## Conclusion

The study provides adequate support for the reliability and validity of the Greek version of MSQv2.1, supporting its potential use in Greek speaking migraineurs and offering a valuable tool for clinical practice and research in the Greek population.

### Electronic supplementary material

Below is the link to the electronic supplementary material.

Supplementary Material 1

## Data Availability

Not applicable.

## Abbreviations

HRQoL: Health-related quality of life
MSQ v.2.1: Migraine-Specific Quality of Life Questionnaire 2.1
SF-12: The 12-item Health Survey
PCS: Physical component summary score
MCS: Mental component summary
MIDAS: Migraine Disability Assessment Questionnaire
MSQv2.1-GR: Greek version of MSQv2.1
RR: Role Restrictive
RP: Role Preventive
EF: Emotional Function
ICC: Intraclass correlation coefficient
ICHD: International Classification of Headache Disorders
