# Supplementary material for: Validity and reliability of the Greek Migraine-Specific Quality of Life Questionnaire (MSQ Version 2.1-GR)
Source: J Patient Rep Outcomes. 2024 Jul 15;8:72. doi: 10.1186/s41687-024-00762-4 (PMC11250746; doi:10.1186/s41687-024-00762-4)
Supplement: Supplementary file 1 — Supplementary Material 1 [file 41687_2024_762_MOESM1_ESM.docx]

**APPENDIX**

**Greek translation of MSQv2.1**

**ΕΡΩΤΗΜΑΤΟΛΟΓΙΟ ΓΙΑ ΤΗΝ ΗΜΙΚΡΑΝΙΑ ΚΑΙ ΤΗΝ ΠΟΙΟΤΗΤΑ ΤΗΣ ΖΩΗΣ^©^**

**(ΕΚΔΟΣΗ 2.1)**

**(Greek translation of the MSQ)**

**ΟΔΗΓΙΕΣ ΓΙΑ ΤΟΝ/ΤΗΝ**

**ΑΣΘΕΝΗ:**

**Παρακαλούμε συμπληρώστε αυτό το ερωτηματολόγιο. Θα μας βοηθήσει να καταλάβουμε τις συνέπειες που έχουν οι ημικρανίες στις καθημερινές σας δραστηριότητες.**

**Το ερωτηματολόγιο έχει σχεδιαστεί έτσι ώστε να μπορεί να συμπληρωθεί γρήγορα και εύκολα. Παρακαλούμε τσεκάρετε μόνο μία απάντηση για κάθε ερώτηση. Θα πρέπει να απαντήσετε σε όλες τις ερωτήσεις.**

**Σας ευχαριστούμε για τη συνεργασία σας.**

Όσο απαντάτε τις παρακάτω ερωτήσεις, σας παρακαλούμε να σκέφτεστε ***όλες τις κρίσεις ημικρανίας*** που μπορεί να είχατε κατά το διάστημα ***των 4 τελευταίων εβδομάδων***.

1. Κατά το διάστημα των 4 τελευταίων εβδομάδων, πόσο συχνά οι ημικρανίες **επηρέασαν** τις σχέσεις σας με την οικογένεια, τους φίλους σας και άλλα πρόσωπα που αισθάνεστε κοντά σας; (επιλέξατε μόνο **μία** απάντηση)

- 1. 🞎 Καθόλου
  2. 🞎 Μικρό διάστημα
  3. 🞎 Μερικές φορές
  4. 🞎 Σημαντικό διάστημα
  5. 🞎 Το μεγαλύτερο διάστημα
  6. 🞎 Συνεχώς

1. Κατά το διάστημα των 4 τελευταίων εβδομάδων, πόσο συχνά οι ημικρανίες **επηρέασαν** τις δραστηριότητες του ελεύθερου χρόνου σας, όπως το διάβασμα ή την άσκηση;

(επιλέξατε μόνο **μία** απάντηση)

- 1. 🞎 Καθόλου
  2. 🞎 Μικρό διάστημα
  3. 🞎 Μερικές φορές
  4. 🞎 Σημαντικό διάστημα
  5. 🞎 Το μεγαλύτερο διάστημα
  6. 🞎 Συνεχώς

1. Κατά το διάστημα των 4 τελευταίων εβδομάδων, πόσο συχνά είχατε **δυσκολία** να κάνετε τη δουλειά σας ή τις καθημερινές σας δραστηριότητες λόγω της ημικρανίας σας;

(επιλέξατε μόνο **μία** απάντηση)

- 1. 🞎 Καθόλου
  2. 🞎 Μικρό διάστημα
  3. 🞎 Μερικές φορές
  4. 🞎 Σημαντικό διάστημα
  5. 🞎 Το μεγαλύτερο διάστημα
  6. 🞎 Συνεχώς

1. Κατά το διάστημα των 4 τελευταίων εβδομάδων, πόσο συχνά οι ημικρανίες σας
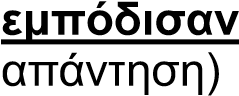
 να κάνετε όσα συνήθως κάνετε στη δουλειά ή στο σπίτι; (επιλέξατε μόνο **μία**

- 1. 🞎 Καθόλου
  2. 🞎 Μικρό διάστημα
  3. 🞎 Μερικές φορές
  4. 🞎 Σημαντικό διάστημα
  5. 🞎 Το μεγαλύτερο διάστημα
  6. 🞎 Συνεχώς

1. Κατά το διάστημα των 4 τελευταίων εβδομάδων, πόσο συχνά οι ημικρανίες **περιόρισαν** την ικανότητά σας να συγκεντρώνεστε στη δουλειά σας ή στις καθημερινές σας δραστηριότητες; (επιλέξατε μόνο **μία** απάντηση)

- 1. 🞎 Καθόλου
  2. 🞎 Μικρό διάστημα
  3. 🞎 Μερικές φορές
  4. 🞎 Σημαντικό διάστημα
  5. 🞎 Το μεγαλύτερο διάστημα
  6. 🞎 Συνεχώς

1. Κατά το διάστημα των 4 τελευταίων εβδομάδων, πόσο συχνά νιώσατε **πολύ κουρασμένος/η** για να κάνετε τη δουλειά σας ή τις καθημερινές σας δραστηριότητες λόγω των ημικρανιών σας; (επιλέξατε μόνο **μία** απάντηση)

- 1. 🞎 Καθόλου
  2. 🞎 Μικρό διάστημα
  3. 🞎 Μερικές φορές
  4. 🞎 Σημαντικό διάστημα
  5. 🞎 Το μεγαλύτερο διάστημα
  6. 🞎 Συνεχώς

1. Κατά το διάστημα των 4 τελευταίων εβδομάδων, πόσο συχνά οι ημικρανίες σας **έκαναν** να νιώθετε λιγότερη ενεργητικότητα; (επιλέξατε μόνο **μία** απάντηση)

- 1. 🞎 Καθόλου
  2. 🞎 Μικρό διάστημα
  3. 🞎 Μερικές φορές
  4. 🞎 Σημαντικό διάστημα
  5. 🞎 Το μεγαλύτερο διάστημα
  6. 🞎 Συνεχώς

1. Κατά το διάστημα των 4 τελευταίων εβδομάδων, πόσο συχνά χρειάστηκε να **ακυρώσετε** τη δουλειά σας ή τις καθημερινές σας δραστηριότητες επειδή είχατε μία ημικρανία;

(επιλέξατε μόνο **μία** απάντηση)

- 1. 🞎 Καθόλου
  2. 🞎 Μικρό διάστημα
  3. 🞎 Μερικές φορές
  4. 🞎 Σημαντικό διάστημα
  5. 🞎 Το μεγαλύτερο διάστημα
  6. 🞎 Συνεχώς

1. Κατά το διάστημα των 4 τελευταίων εβδομάδων, πόσο συχνά **χρειαστήκατε βοήθεια** για να διεκπεραιώσετε συνηθισμένες υποχρεώσεις όπως το καθημερινό νοικοκυριό, την εξόφληση λογαριασμών, τα ψώνια, ή να φροντίζετε άλλους, όταν είχατε μία ημικρανία;

(επιλέξατε μόνο **μία** απάντηση)

- 1. 🞎 Καθόλου
  2. 🞎 Μικρό διάστημα
  3. 🞎 Μερικές φορές
  4. 🞎 Σημαντικό διάστημα
  5. 🞎 Το μεγαλύτερο διάστημα
  6. 🞎 Συνεχώς

1. Κατά το διάστημα των 4 τελευταίων εβδομάδων, πόσο συχνά χρειάστηκε **να σταματήσετε** τη δουλειά σας ή τις καθημερινές σας υποχρεώσεις για να αντιμετωπίσετε τα συμπτώματα της ημικρανίας; (επιλέξατε μόνο **μία** απάντηση)

- 1. 🞎 Καθόλου
  2. 🞎 Μικρό διάστημα
  3. 🞎 Μερικές φορές
  4. 🞎 Σημαντικό διάστημα
  5. 🞎 Το μεγαλύτερο διάστημα
  6. 🞎 Συνεχώς

1. Κατά το διάστημα των 4 τελευταίων εβδομάδων, πόσο συχνά **ήσαστε ανίκανοι να συμμετάσχετε** σε κοινωνικές δραστηριότητες όπως σε πάρτυ, σε τραπέζι με φίλους, κ.λ.π., επειδή είχατε μία ημικρανία; (επιλέξατε μόνο **μία** απάντηση)

- 1. 🞎 Καθόλου
  2. 🞎 Μικρό διάστημα
  3. 🞎 Μερικές φορές
  4. 🞎 Σημαντικό διάστημα
  5. 🞎 Το μεγαλύτερο διάστημα
  6. 🞎 Συνεχώς

1. Κατά το διάστημα των 4 τελευταίων εβδομάδων, πόσο συχνά **νιώσατε** μπουχτισμένος/η ή εκνευρισμένος/η λόγω των ημικρανιών σας; (επιλέξατε μόνο **μία** απάντηση)

- 1. 🞎 Καθόλου
  2. 🞎 Μικρό διάστημα
  3. 🞎 Μερικές φορές
  4. 🞎 Σημαντικό διάστημα
  5. 🞎 Το μεγαλύτερο διάστημα
  6. 🞎 Συνεχώς

1. Κατά το διάστημα των 4 τελευταίων εβδομάδων, πόσο συχνά **νιώσατε** σαν να ήσαστε ένα βάρος στους άλλους λόγω των ημικρανιών σας; (επιλέξατε μόνο **μία** απάντηση)

- 1. 🞎 Καθόλου
  2. 🞎 Μικρό διάστημα
  3. 🞎 Μερικές φορές
  4. 🞎 Σημαντικό διάστημα
  5. 🞎 Το μεγαλύτερο διάστημα
  6. 🞎 Συνεχώς

1. Κατά το διάστημα των 4 τελευταίων εβδομάδων, πόσο συχνά **φοβηθήκατε** μήπως απογοητεύσετε τους άλλους λόγω των ημικρανιών σας; (επιλέξατε μόνο **μία** απάντηση)

- 1. 🞎 Καθόλου
  2. 🞎 Μικρό διάστημα
  3. 🞎 Μερικές φορές
  4. 🞎 Σημαντικό διάστημα
  5. 🞎 Το μεγαλύτερο διάστημα
  6. 🞎 Συνεχώς
